# Thermoregulatory effects of guava leaf extract-menthol toner application for post-exercise use

**DOI:** 10.1080/13880209.2021.1942925

**Published:** 2021-07-01

**Authors:** Titeyut Wongsanao, Wipavadee Leemingsawat, Vipaporn Panapisal, Thanomwong Kritpet

**Affiliations:** a Faculty of Sports Science, Chulalongkorn University, Bangkok, Thailand; b Department of Pharmaceutics and Industrial Pharmacy and Cosmetics Strategic Research Unit, Chulalongkorn University Drug and Health Products Innovation & Promotion Center, Faculty of Pharmaceutical Sciences, Chulalongkorn University, Bangkok, Thailand

**Keywords:** Astringent, sweating, skin temperature, heat dissipation, personal hygiene

## Abstract

**Context:**

*Psidium guajava* L. (Myrtaceae) leaf contains a wide variety of bioactive compounds that contribute valuable effects on human well-being.

**Objective:**

This study investigates the influence of guava leaf extract-menthol toner on thermoregulation, including perspiration, skin temperature, and recovery heart rate.

**Materials and methods:**

This randomised, placebo-controlled clinical trial assessed the effects of the guava leaf extract-menthol toner and placebo with a 1-week washout period. Sixty-four participants were enrolled. The participants exercised on a treadmill until a 75% heart rate reserve was achieved for 5 min, followed by a 5 min post-exercise rest period. The skin temperature and heart rate were then measured before 5 mL of the testing product was sprayed to specific areas of the body, left it for 30 sec before wiped off. Post-exercise perspiration and skin temperatures were collected by sweat patches and measured by the Skin-thermometer ST500, respectively. A 20 min heart rate monitoring period started 10 min after the exercise and measured every 2 min intervals.

**Results:**

Use of the toner significantly reduced post-exercise perspiration to approximately half of the baseline and placebo use values (*p* < 0.05). Furthermore, relative heart rate changes showed no significant differences among the tests (*p* > 0.05). Skin temperature was also unaffected (*p* > 0.05).

**Discussion and Conclusion:**

Guava leaf extract-menthol toner reduced perspiration by astringent effects but did not influence heat dissipation and did not affect cardiovascular mechanism compared to the controls. Additional cleaning with guava leaf extract-menthol toner could offer better hygiene after a workout.

## Introduction

Thermoregulatory and cardiovascular processes, including skin blood flow and sweating, during sustained exercise are significantly associated with increased body heat content due to metabolic heat production (Kenny and McGinn 2017). Sweating mainly reduces core and skin temperature during exercise (Tansey and Johnson 2015); then at the cessation of exercise, sweat production rate decreases rapidly, and the principle route of heat dissipation is likely convection through the skin in the period following exercise (Gerrett et al. 2018). However, sweat alkalinity during excessive perspiration resulting from bicarbonate ions (HCO_3_^-^) might alter the skin barrier and cause various skin diseases (Schmid-Wendtner and Korting 2006). Sweating from exercise was reported as the common factor which aggravated the symptoms of atopic dermatitis in school children (12–14 year-olds) (Williams et al. 2004). Moreover, secretion of sweat and leakage into tissues could promote itching for people with atopic dermatitis and aggravate dermatitis (Murota et al. 2018). Additionally, apocrine sweat glands secrete milk-like substances composed of electrolytes, steroids, proteins, vitamins, and lipid compounds, which might be transformed by skin flora bacteria and cause body odour (Fredrich et al. 2013). Therefore, post-exercise hygienic practices should quickly clean sweat stains from the skin, and if there is a way to decrease the remaining post-exercise perspiration, then the occurrence of the aforementioned problems will be prevented. Since the restoration of thermoregulation after exercise turns to be primarily convection through the skin instead, the body is still recovering after a workout.

Guava leaves [*Psidium guajava* L. (Myrtaceae)], which contain phytochemical phenolic compounds (Morais-Braga et al. 2017), are regionally used as an alternative medicine for disinfecting the human gastrointestinal tract (Daswani et al. 2011; de Souza et al. 2014). Moreover, guava leaf extracts have astringent properties resulting from tannin phenolics (Ashok and Upadhyaya 2012) and can be utilised as a skin toner for tightening skin pores and decreasing oily skin (Pongsakornpaisan et al. 2019). Further, menthol, at various concentrations, is generally used as a cosmetic cooling agent in healthcare products (Kamatou et al. 2013). Currently, it is known that applying menthol can excite the transient receptor potential melastatin 8 (TRPM8) that can cause cutaneous vasodilation (Craighead and Alexander 2016) and increase the process of convection heat dissipation through the skin (Wong and Hollowed 2017). To date, it remains unknown whether the proven astringent effects of guava leaf extract and cutaneous vasodilation, and heat-convection of menthol can be extrapolated during the post-exercise recovery period. Specifically, there is inadequate research on the utilisation of both substances to develop alternative skincare and healthcare products suitable for post-exercise application to resolve problems associated with post-exercise perspiration without affecting heat dissipation.

Therefore, this study aimed to investigate the influence of guava leaf extract-menthol toner (GMT) for post-exercise use on thermoregulatory effects including perspiration and skin temperature as well as recovery heart rate compared with that of the placebo (PCB) and baseline (BSL) values. It was hypothesised that the GMT toner could remarkably reduce perspiration after sweat stain cleaning due to the astringent properties of guava leaf extract. Nonetheless, exercise recovery, including the relative changes of heart rate (HR) and skin temperature reductions, should not be altered because the heat dissipation primarily goes on with the heat convection due to the TRPM8 activation of menthol which causing cutaneous vasodilation. These findings may potentially be helpful based on the potential future use of GMT to clean sweat stains, reduce post-exercise perspiration, and enhance post-exercise body heat dissipation, as well as allowing for better hygiene for individuals who exercise or those with hyperhidrosis before regular bathing.

## Materials and methods

### Materials

Commercial guava leaf extract was purchased from Specialty Natural Products Co., Ltd., Chonburi, Thailand (Lot No. HE-EL41-PSG) and quantified for the total phenolic and flavonoid contents at the Faculty of Pharmaceutical Sciences, Chulalongkorn University (Bangkok, Thailand). The GMT formulation contains 20% v/v of guava leaf extract, 1% w/v of menthol (1381709 USP, Sigma-Aldrich Pte. Ltd., Singapore), aluminium chlorohydrate (ICL performance products Ltd., China), ethanol (RCI Labscan Ltd., Thailand), Polysorbate 80 (Spectrum chemical Mfg., Corp., USA), perfumes, and purified water. The placebo formulation (PCB) composes the same toner base without the actives (i.e., guava leaf extract and menthol). All chemicals are either pharmaceutical or cosmetic grade. The studied preparations were prepared and evaluated for physical and chemical stabilities at the compounding laboratory, the Faculty of Pharmaceutical Sciences, Chulalongkorn University (Bangkok, Thailand).

### Ethical approval

This study was approved by the Research Ethics Review Committee, Health Science Groups, Chulalongkorn University, Thailand (COA No. 176/2017), and was performed following the Declaration of Helsinki. Prior to the commencement of the experiments, the participants were informed about the procedures. Thereafter, a signed consent form for study participation was collected from each participant.

### Participants

Healthy participants, consisting of 23 men and 41 women (age range: 18–24 years), were enrolled in this study. All participants underwent assessment for readiness to exercise using the Physical Activity Readiness Questionnaire (PAR-Q) (Shephard 2015; Whitfield et al. 2017), and did not have a history of severe skin allergy to any topical products requiring treatment.

### Preliminary testing

One week prior to the main trials, the participants visited the laboratory where they were initially familiarised with the experimental protocol and equipment and were tested to identify individual treadmill speed and slope that properly allowed aerobic exercise. Briefly, the individual’s 75% HR reserve (HRR) was calculated by applying Karvonen's formula (Camarda et al. 2008). Each participant exercised by running on the treadmill (Mercury^®^ med, H/P/Cosmos Sports & Medical Gmbh, Nußdorf, Germany) with a warm-up speed of 2.7 km/h and a 10% slope for 3 min. Subsequently, each participant performed an incremental Modified Bruce protocol (Stefani et al. 2010) with increments of speed by 1.3 km/h and slope by 2% grade every 3 min to approximately 75% individual HRR (±5 beats/min). The speed and slope of the treadmill were maintained for 5 min followed by a cooling down period for 5 min. At this stage, participants who were unable to achieve a vigorous-intensity exercise at the assigned workload and time duration or to produce sufficient sweat were excluded. Furthermore, the mean speed and constant slope of the treadmill to preserve the 75% individual HRR were fixed for each participant during the main trials.

### Main trial

**Figure 1.**
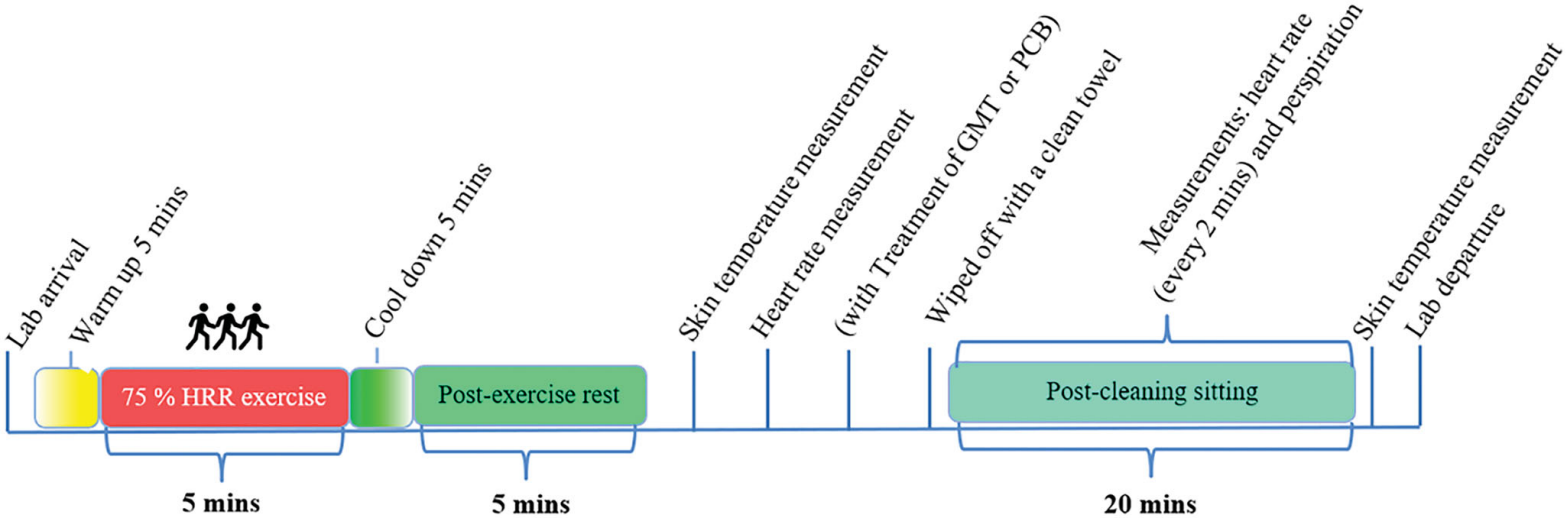
Time course of the experimental procedure.

All experiments were conducted during the summer (between March and April) and at the same time of the day. For 24 h before their first experimental trial, the participants were instructed not to exercise, consume high sodium (salt) content foods, or drink alcohol or coffee to ensure similar levels of nourishment and hydration. Further, a good night’s sleep was recommended before the experiments, and participants were advised to have food and drink at least 2 h and 1 h prior to the trials, respectively. These instructions were replicated before each experiment. All of the participants attended the laboratory at the same time of the day, to avoid circadian variation in skin temperature, on three separate occasions with a one-week interval. On the first experiment day, the baseline test without any treatment (BSL), all participants underwent acclimatisation to the test facility with the controlled conditions: 25 ± 2 °C and 60 ± 2% RH for 30 min. The test began with a 5 min warm-up session involving walking on a treadmill at a speed of 3.0 km/h and a 14% slope. Subsequently, the participants were instructed to exercise at the previously determined speed and slope to reach an exercise intensity of 75% HRR for 5 min. Then the participants cooled down for 5 min and rested for 5 min; then the skin temperature and HR measurements were taken. Before the post-exercise monitoring period, the participants cleaned their whole body with a clean towel to remove sweat stains. Perspiration was collected by attached sweat patches on different body parts of each participant’s skin while they were sitting in the study room for 20 min. Simultaneously, the recovery HR was measured at 2 min intervals till the end of 20 min, and the skin temperature was again measured at the end of the period (Figure 1).

A single-blind, randomised, placebo-controlled crossover trial was used in this study. On the other two separate experiment days, the same subjects were randomly assigned to groups that received alternative sequences of treatments (i.e., GMT followed by PCB (GMT-PCB) or PCB followed by GMT (PCB-GMT)) with a one-week washout period between them. On each occasion, participants performed the same procedure as in the BSL test; however, 5 mL of GMT or PCB was sprayed on the chin, neck, chest, body, armpits, arms, and legs after obtaining the initial skin temperature and HR measurements and left for 30 s. Next, the whole body was cleaned with a towel until no moisture was left before proceeding to the next step.

### Measurements

#### Post-exercise perspiration

The post-exercise perspiration was measured during the 20 min sitting period after thorough removal of sweat stains by wiping with a clean towel after the BSL test or by using either GMT or PCB toner and then wiping with a clean towel. This was achieved by fixing 200 cm^2^ weighed sweat patches (SOS Plus, Wayson Medical, Thailand) on the back of the forearms, back, chest, and upper thighs. Further, a 42 cm^2^ weighted sweat patch was placed on the forehead (Baker et al. 2009). At the end of the 20 min sitting, all the sweat patches were removed and weighed using the same balance (TE313S-DS, 310 g × 0.001 g, Sartorius, Germany). The before-after weight difference was calculated for post-exercise perspiration.

#### Recovery heart rate

The first HR measurement was obtained in the post-exercise recovery stage (Polar A300 with heart rate Sensor, Polar Electro, Finland) at the end of the 5 min resting period after exercise. Subsequently, HR measurements were obtained at 2 min intervals for 20 min during the post-exercise monitoring period in each trial of with or without treatments, respectively. Relative changes in HR (ΔHR) during the 20 min resting period were calculated.

#### Skin temperature

The skin temperatures (Skin-thermometer ST500, Courage + Khazaka electronic GmbH, Germany) were measured at the end of the 5 min post-exercise resting period and after the 20 min sitting period. Skin temperatures were measured at the forehead, chest, dorsal aspect of forearms, thighs, calves, lower abdomen, and back of the participants (Powers and Howley 2012). The mean skin temperature of all measured positions was reported.

### Statistical analysis

All data analyses were conducted using the SPSS version 23 program (IBM Corporation, USA). Initially, the data of each parameter were examined for variance homogeneity to assume the variances were either homogeneous or heterogeneous, using a particular analysis of variance (ANOVA) measurements to analyse the mean differences. Later, repeated two-way ANOVA (3 × 9: group × time pair) was used for between-group comparisons of the relative changes in HR (ΔHR) during the 20 min sitting period. To evaluate differences between groups in the recovery HRs after the 5 min post-exercise resting period and the changes between skin temperatures after the 5 min post-exercise rest and the 20 min post-cleaning period, one-way ANOVA for between-group comparisons was used. After a significant F value, the Bonferroni *post hoc* tests were conducted to identify pairwise differences. Due to heterogeneous variance data, one-way ANOVA utilising the Brown-Forsythe method to examine the mean equality followed by the Games-Howell *post hoc* tests were used to identify significant post-exercise perspiration pairwise differences. Significant differences were determined at *p* < 0.05. Data are presented as the mean ± standard deviation.

## Results

Preliminary stability data revealed that the light brown clear GMT toner was chemically and physically stable when stored in a light-protected container at room temperature for at least 6 months (data not shown). Moreover, GMT was reported to pass an allergic skin test upon examination in 40 male and female volunteers.

There were no unusual occurrences regarding the examined products and experimental designs for all the enrolled 64 participants (Table 1). The average age of the participants was 19.6 ± 1.1 years with a weight and height range of 47.4–73.2 kg and 158.5–175.3 cm, respectively, and an average resting HR range of 66–90 beats/min. All participants had no record of skin product allergy and passed the pre-exercise readiness assessment using the PAR-Q questionnaires.

**Table 1.** Descriptive characteristics of the participants.

Characteristics	Male (*n* = 23)	Female (*n* = 41)	Total (*n* = 64)
Age (years)	20.0 ± 1.3	19.4 ± 1.0	19.6 ± 1.1
Weight (kg)	70.0 ± 12.6	54.8 ± 9.4	60.3 ± 12.9
Height (cm)	175.1 ± 6.3	162.3 ± 5.5	166.9 ± 8.4
RSBP (mm Hg)	122.1 ± 10.4	106.0 ± 10.0	111.8 ± 12.7
RDBP (mm Hg)	76.3 ± 5.6	69.8 ± 5.9	72.1 ± 6.6
RHR (beats/min)	72 ± 12	81 ± 11	78 ± 12

Values are presented as mean ± SD. RSBP, resting systolic blood pressure; RDBP, resting diastolic blood pressure; RHR, resting heart rate.

### Perspiration or sweating amounts in the post-exercise stage

Post-exercise perspiration in the GMT test was reduced by about half when compared with that in PCB and BSL experiments, whereas the PCB test showed insignificantly different results from the BSL values. Table 2 presents the post-exercise perspiration in all the tests.

**Table 2.** The heart rate after the 5 min post-exercise resting (HR), the post-exercise perspiration after the 20 min post-cleaning (PR), and the changes in the skin temperature following the 20 min post-cleaning (T_sk_) of all the participants in each test (*n* = 64).

Groups	GMT	PCB	BSL	*p* value
PR (mL)	0.1 (0.1)	0.2 (0.2)	0.2 (0.3)	0.02^a^^,b^
HR (beats/min)	100 (10)	101 (11)	104 (12)	0.10
T_sk_ (°C)	0.9 (0.8)	1.0 (0.8)	0.9 (0.5)	0.38

Values are presented as mean (SD). GMT, guava leaf extract-menthol toner; PCB, placebo; BSL, baseline.

^a^Significant difference (*p* < 0.05) between GMT and PCB; ^b^Significant difference (*p* < 0.05) between GMT and BSL.

### Skin temperature characteristics

After the 5 min post-exercise resting period, skin temperatures in all trials were approximately one degree Celsius lower than those before exercise (data not shown). The skin temperatures tended to rise after the 20 min post-cleaning sitting period; the PCB test showed the highest skin temperature change, while the GMT and BSL tests showed comparable changes (as shown in Table 2). Nevertheless, the changes in the skin temperature following the 20 min post-cleaning (Tsk) were statistically insignificant (*p* = 0.38) for all the tests.

### Recovery heart rate responses

**Figure 2.**
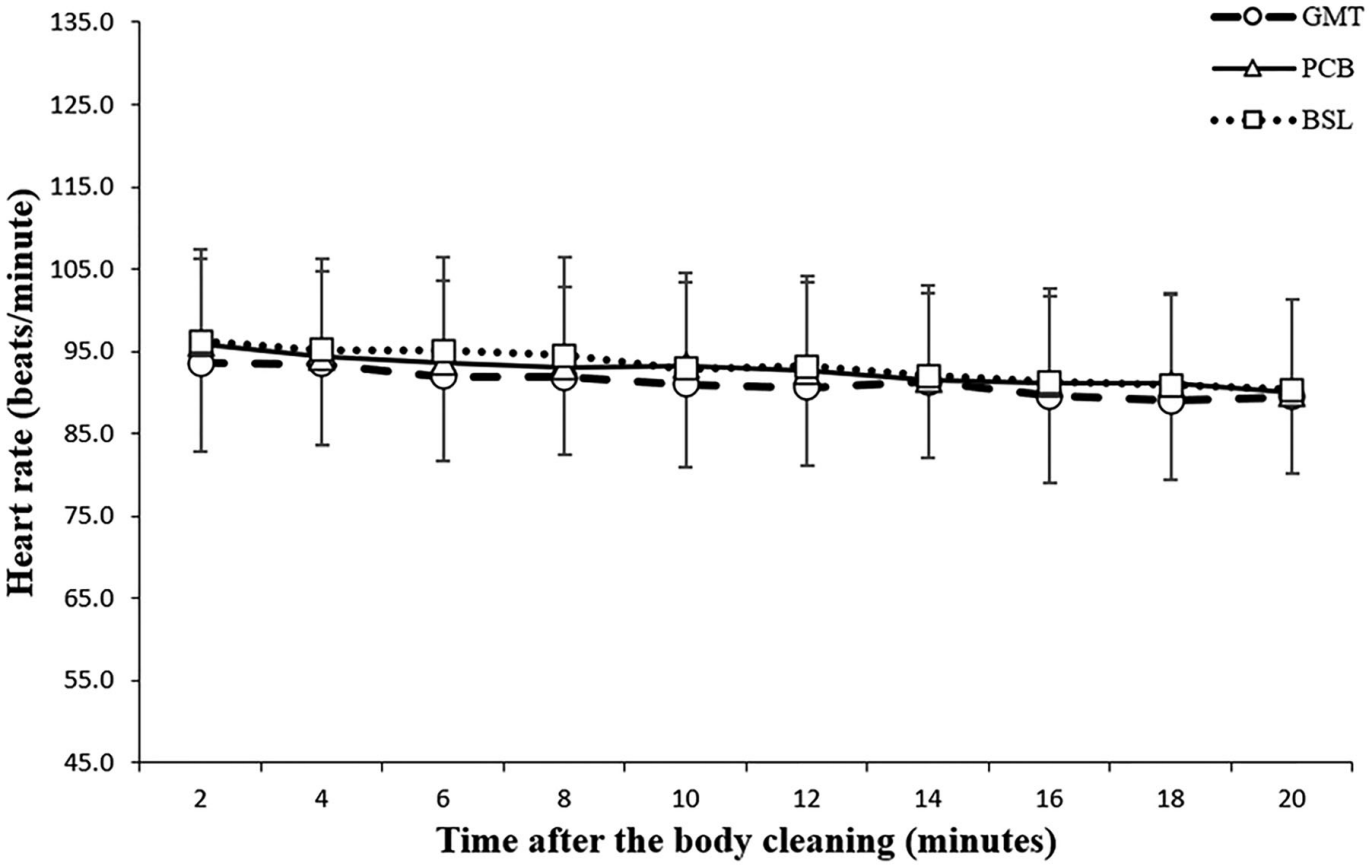
The heart rate (HR) of all the participants (*n* = 64) after the guava leaf extract-menthol toner (GMT), placebo (PCB), and baseline (BSL) tests. Consecutive measurements were performed at 2 min intervals in the 20 min post-cleaning period. (No significant differences existed between groups; *p* = 0.12).

As shown in Table 2, the recovery HRs obtained at the end of the 5 min post-exercise resting period were comparable among all the three tests (*p* = 0.10). Figure 2 also shows comparative declining curves of the recovery HR in each experiment. Furthermore, as shown in Table 3, between-group comparisons of the relative changes in HR (ΔHR) during the 20 min post-exercise monitoring period after complete cleaning showed no significant difference among all the three tests (*p* = 0.39).

**Table 3.** The changes in post-exercise heart rates compared with the 2 min post-cleaning heart rate in all the participants during each group measured at 2 min intervals within the 20 min post-cleaning period (*n* = 64).

	Relative heart rates (beats/min)		
	GMT	PCB	BSL
Change intervals			
Δ4–2 min	−0.2 (5.8)	−1.6 (3.9)	−1.1 (4.3)
Δ6–2 min	−1.6 (5.6)	−2.2 (4.5)	−1.1 (5.3)
Δ8–2 min	−1.7 (6.5)	−2.8 (4.3)	−1.7 (4.4)
Δ10–2 min	−2.6 (6.1)	−2.7 (5.6)	−3.3 (5.2)
Δ12–2 min	−3.0 (5.0)	−3.3 (5.5)	−3.1 (4.7)
Δ14–2 min	−2.3 (5.9)	−4.4 (6.1)	−4.2 (5.2)
Δ16–2 min	−4.0 (6.0)	−4.8 (5.8)	−4.9 (4.9)
Δ18–2 min	−4.6 (6.5)	−4.8 (5.7)	−5.3 (5.0)
Δ20–2 min	−4.2 (6.4)	−6.0 (6.0)	−5.8 (4.1)
Between-group comparisons	*p* value		
Group	0.39^a^		
Time	<0.00^b^		
Group × Time	0.35^c^		

Values are presented as mean (SD). GMT, guava leaf extract-menthol toner; PCB, placebo; BSL, baseline.

^a^No significant interaction existed between groups (*p* > 0.05); ^b^Significant between-group difference (*p* < 0.05); ^c^No significant interaction existed between groups and time (*p* > 0.05).

## Discussion

During post-workout recovery, the routine should generally include a cool-down period before you take a shower, but if time does not allow for it then the use of a natural extract toner would offer an extra benefit including a reduction in perspiration, antibacterial, etc. The main findings indicate that GMT efficiently decreased the post-exercise perspiration during resting without significantly altering the body recovery after physical exercise. The efficient decrease in post-exercise perspiration could be attributed to the properties of guava leaf extract. Previous studies have shown that guava leaf extract contains tannins (Biswas et al. 2013; Mailoa et al. 2014), which have astringent properties capable of perspiration inhibition. The anti-perspiration effect of tannins might involve allowing denaturation of superficial layer proteins in the stratum corneum and obstruction of the sweat pores, which reduces the rate of perspiration (Piérard et al. 2003). Moreover, another reason for GMT to decrease perspiration greater than PCB and BSL was likely due to some constituents in the formulation. Guava leaf extract, the main ingredient in the GMT, contains glycerine as a solvent. Glycerine is well known as an efficient humectant and has an excellent hygroscopic property by which it can hydrate the stratum corneum and make it swell (Leelapornpisid et al. 2014). The mechanism of skin moisturisation by glycerine is probably by drawing water from other epidermis layers to the stratum corneum; additionally, the glycerine film could prevent evaporation of sweat from the skin (Flynn et al. 2001; Rawlings et al. 2004). Therefore, the occlusive mechanism of glycerine in the guava leaf extract on the skin may have caused a significant decrease in perspiration observed with GMT application compared to that of BSL and PCB tests.

Moreover, the GMT formulation contains another active substance, menthol, which stimulates the TRPM8 channel expressed in a subpopulation of sensory neurons and is also activated by cold sensation. Besides, this receptor also regulates conscious human skin blood flow (Johnson et al. 2009). Previous study has shown that applying menthol could also excite this receptor and could cause cutaneous vasodilation (Craighead and Alexander 2016). Further, menthol could stimulate TRPM8 on the aorta smooth muscle, causing the decreases of extracellular and intracellular calcium ions, resulting in smooth muscle relaxation from depolarisation and causing vasodilation (Yang et al. 2006). The cutaneous vasodilation may have a beneficial effect on the heat dissipation of the body because it may increase the heat dissipation from the core body to the environment by convection via the skin (Wong and Hollowed 2017).

The Skin-Thermometer ST500 equipment is equipped with a special sensor in which the measurement is based on contact-free infra-red recognition of heat emission from the skin. Skin temperature is defined by its microcirculation; the more the circulation at a certain area on the skin, the higher the skin temperature. During the recovery period after exercise, evaporation of sweat and increased skin blood flow are effective mechanisms for heat dissipation from the body. The slightly elevated skin temperatures after the 20 min post-cleaning period may relate to the convection heat loss (Torii et al. 1992). The current findings revealed that the changes in the skin temperature following the 20 min post-cleaning (Tsk) showed no significant differences.

The novel formulated GMT toner contains guava leaf extract and menthol, which reduced perspiration by astringent effects and affected cutaneous vasodilation by TRPM8 receptor activation, respectively. Even though sweating is mainly a primary means of cooling during exercise; on cessation of exercise, it is likely that the main route of heat loss is convection from the skin (Gerrett et al. 2018). Therefore, GMT toner could alleviate skin problems causing by sweat strain and promote heat dissipation through convection. Moreover, the changes in HR during the 20 min rest showed comparable reductions in all tests, and the relative changes (ΔHR) were not significantly different among the tests, which could imply that the reduction in perspiration or cutaneous vasodilation of GMT did not significantly alter the body recovery process.

There is a global acceptance that physical activity is an important component of a healthy lifestyle in children and adolescents. Physical education programs have been modified to increase more class period and frequency engaging in moderate-to-vigorous physical activity. As a result, there is no time to shower before moving on to the next class. Our findings implied that the topical application of guava leaf extract-menthol toner could reduce post-exercise perspiration and facilitate cleaning of sweat strain. In this study, considering that most of our participants are adolescents involved in a vigorous-intensity exercise, which is usually in regular physical education program, applying GMT toner for the primary cleaning before taking a shower could be suggested for better personal hygiene. Additional investigation on antibacterial properties would fulfil the benefits of this natural toner. However, an assessment of antibacterial capability should be properly designed in future study to conform to briefly apply of the product and wipe off.

## Conclusions

Sweat strain after a workout could adversely affect the skin. Further, bacteria might transform sweat stain accumulated on the skin into body odour. GMT application decreased post-exercise perspiration; therefore, it may resolve problems associated with excessive sweat on the skin, especially when there is no time for regular body cleaning. The current findings showed that lowered post-exercise perspiration did not influence heat dissipation and did not affect cardiovascular mechanism compared to baseline and placebo. In summary, natural GMT could offer an alternative means for improving post-exercise self-hygienic practices. In addition, it may alleviate skin problems associated with sweat strain commonly seen nowadays in our busy lifestyle.

## References

[CIT0001] Ashok PK, Upadhyaya K. 2012. Tannins are astringent. J Pharmacogn Phytochem. 1(3):45–50.

[CIT0002] Baker LB, Stofan JR, Hamilton AA, Horswill CA. 2009. Comparison of regional patch collection vs. whole body washdown for measuring sweat sodium and potassium loss during exercise. J Appl Physiol. 107(3):887–895. 19541738 10.1152/japplphysiol.00197.2009

[CIT0003] Biswas B, Rogers K, McLaughlin F, Daniels D, Yadav A. 2013. Antimicrobial activities of leaf extracts of guava (*Psidium guajava* L.) on two Gram-negative and Gram-positive bacteria. Int J Microbiol. 2013:746165. 24223039 10.1155/2013/746165 PMC3817707

[CIT0004] Camarda SR, Tebexreni AS, Páfaro CN, Sasai FB, Tambeiro VL, Juliano Y, Barros Neto TL. 2008. Comparison of maximal heart rate using the prediction equations proposed by Karvonen and Tanaka. Arq Bras Cardiol. 91(5):311–314. 19142375 10.1590/s0066-782x2008001700005

[CIT0005] Craighead DH, Alexander LM. 2016. Topical menthol increases cutaneous blood flow. Microvasc Res. 107:39–45. 27131832 10.1016/j.mvr.2016.04.010 PMC5406845

[CIT0006] Daswani PG, Ghadge AA, B S, Birdi TJ. 2011. Preparation of decoction of medicinal plants: a self-help measure? J Altern Complement Med. 17(12):1099–1100. 22106933 10.1089/acm.2011.0217 PMC3241206

[CIT0007] de Souza F, Parker T, Ali A. 2014. Exploring the utility of *Psidium guajava* leaf extract as an adequate treatment for *Giardia lamblia*. J Altern Complement Med. 20(5):A72–A72.

[CIT0008] Flynn TC, Petros J, Clark RE, Viehman GE. 2001. Dry skin and moisturizers. Clin Dermatol. 19(4):387–392. 11535378 10.1016/s0738-081x(01)00199-7

[CIT0009] Fredrich E, Barzantny H, Brune I, Tauch A. 2013. Daily battle against body odor: towards the activity of the axillary microbiota. Trends Microbiol. 21(6):305–312. 23566668 10.1016/j.tim.2013.03.002

[CIT0010] Gerrett N, Griggs K, Redortier B, Voelcker T, Kondo N, Havenith G. 2018. Sweat from gland to skin surface: production, transport, and skin absorption. J Appl Physiol (1985). 1985. 125(2):459–469. 29745799 10.1152/japplphysiol.00872.2017

[CIT0011] Johnson CD, Melanaphy D, Purse A, Stokesberry SA, Dickson P, Zholos AV. 2009. Transient receptor potential melastatin 8 channel involvement in the regulation of vascular tone. Am J Physiol Heart Circ Physiol. 296(6):H1868–1877. 19363131 10.1152/ajpheart.01112.2008 PMC2716108

[CIT0012] Kamatou GP, Vermaak I, Viljoen AM, Lawrence BM. 2013. Menthol: a simple monoterpene with remarkable biological properties. Phytochemistry. 96:15–25. 24054028 10.1016/j.phytochem.2013.08.005

[CIT0013] Kenny GP, McGinn R. 2017. Restoration of thermoregulation after exercise. J Appl Physiol (1985). 122(4):933–944. 27881668 10.1152/japplphysiol.00517.2016

[CIT0014] Leelapornpisid P, Mungmai L, Sirithunyalug B, Jiranusornkul S, Peerapornpisal Y. 2014. A novel moisturizer extracted from freshwater macroalga [*Rhizoclonium hieroglyphicum* (C.Agardh) K tzing] for skin care cosmetic. Chiang Mai J Sci. 41(5.2):1195–1207.

[CIT0015] Mailoa MN, Mahendradatta M, Laga A, Djide N. 2014. Antimicrobial activities of tannins extract from guava leaves [*Psidium Guajava* L. (Myrtaceae)] on pathogens microbial. Int J Sci Technol Res. 3(1):236–241.

[CIT0016] Morais-Braga MF, Carneiro JN, Machado AJ, Sales DL, Dos Santos AT, Boligon AA, Athayde ML, Menezes IR, Souza DS, Costa JG, et al. 2017. Phenolic composition and medicinal usage of *Psidium guajava* Linn.: antifungal activity or inhibition of virulence? Saudi J Biol Sci. 24(2):302–313. 28149166 10.1016/j.sjbs.2015.09.028 PMC5272930

[CIT0017] Murota H, Yamaga K, Ono E, Katayama I. 2018. Sweat in the pathogenesis of atopic dermatitis. Allergol Int. 67(4):455–459. 30082151 10.1016/j.alit.2018.06.003

[CIT0018] Piérard G, Elsner P, Marks R, Masson P, Paye M. 2003. EEMCO guidance for the efficacy assessment of antiperspirants and deodorants. Skin Pharmacol Appl Skin Physiol. 16(5):324–342. 12907837 10.1159/000072072

[CIT0019] Pongsakornpaisan P, Lourith N, Kanlayavattanakul M. 2019. Anti-sebum efficacy of guava toner: a split-face, randomized, single-blind placebo-controlled study. J Cosmet Dermatol. 18(6):1737–1741. 30964238 10.1111/jocd.12943

[CIT0020] Powers SK, Howley ET. 2012. Exercise physiology: theory and application to fitness and performance. 8th ed. New York (NY): McGraw-Hill.

[CIT0021] Rawlings AV, Canestrari DA, Dobkowski B. 2004. Moisturizer technology versus clinical performance. Dermatol Ther. 17(s1):49–56. 14728699 10.1111/j.1396-0296.2004.04s1006.x

[CIT0022] Schmid-Wendtner MH, Korting HC. 2006. The pH of the skin surface and its impact on the barrier function. Skin Pharmacol Physiol. 19(6):296–302. 16864974 10.1159/000094670

[CIT0023] Shephard RJ. 2015. Qualified fitness and exercise as professionals and exercise prescription: evolution of the PAR-Q and Canadian aerobic fitness test. J Phys Act Health. 12(4):454–461. 24836975 10.1123/jpah.2013-0473

[CIT0024] Stefani L, Mascherini G, Galanti G. 2010. Aerobic threshold for exercise prescription. IJCM. 1(1):6–9.

[CIT0025] Tansey EA, Johnson CD. 2015. Recent advances in thermoregulation. Adv Physiol Educ. 39(3):139–148. 26330029 10.1152/advan.00126.2014

[CIT0026] Torii M, Yamasaki M, Sasaki T, Nakayama H. 1992. Fall in skin temperature of exercising man. Br J Sports Med. 26(1):29–32. 1600450 10.1136/bjsm.26.1.29 PMC1478977

[CIT0027] Whitfield GP, Riebe D, Magal M, Liguori G. 2017. Applying the ACSM preparticipation screening algorithm to U.S. adults: national health and nutrition examination survey 2001–2004. Med Sci Sports Exerc. 49(10):2056–2063. 28557860 10.1249/MSS.0000000000001331 PMC7059860

[CIT0028] Williams JR, Burr ML, Williams HC. 2004. Factors influencing atopic dermatitis-a questionnaire survey of school children's perceptions. Br J Dermatol. 150(6):1154–1161. 15214903 10.1111/j.1365-2133.2004.05869.x

[CIT0029] Wong BJ, Hollowed CG. 2017. Current concepts of active vasodilation in human skin. Temperature (Austin). 4(1):41–59. 28349094 10.1080/23328940.2016.1200203 PMC5356216

[CIT0030] Yang XR, Lin MJ, McIntosh LS, Sham JS. 2006. Functional expression of transient receptor potential melastatin- and vanilloid-related channels in pulmonary arterial and aortic smooth muscle. Am J Physiol Lung Cell Mol Physiol. 290(6):L1267–L1276. 16399784 10.1152/ajplung.00515.2005

